# Supplementary material for: DNA vaccination protects mice against Zika virus-induced damage to the testes
Source: Nat Commun. 2017 Jun 7;8:15743. doi: 10.1038/ncomms15743 (PMC5467228; doi:10.1038/ncomms15743)
Supplement: Supplementary Information — Supplementary Figures [file ncomms15743-s1.pdf]

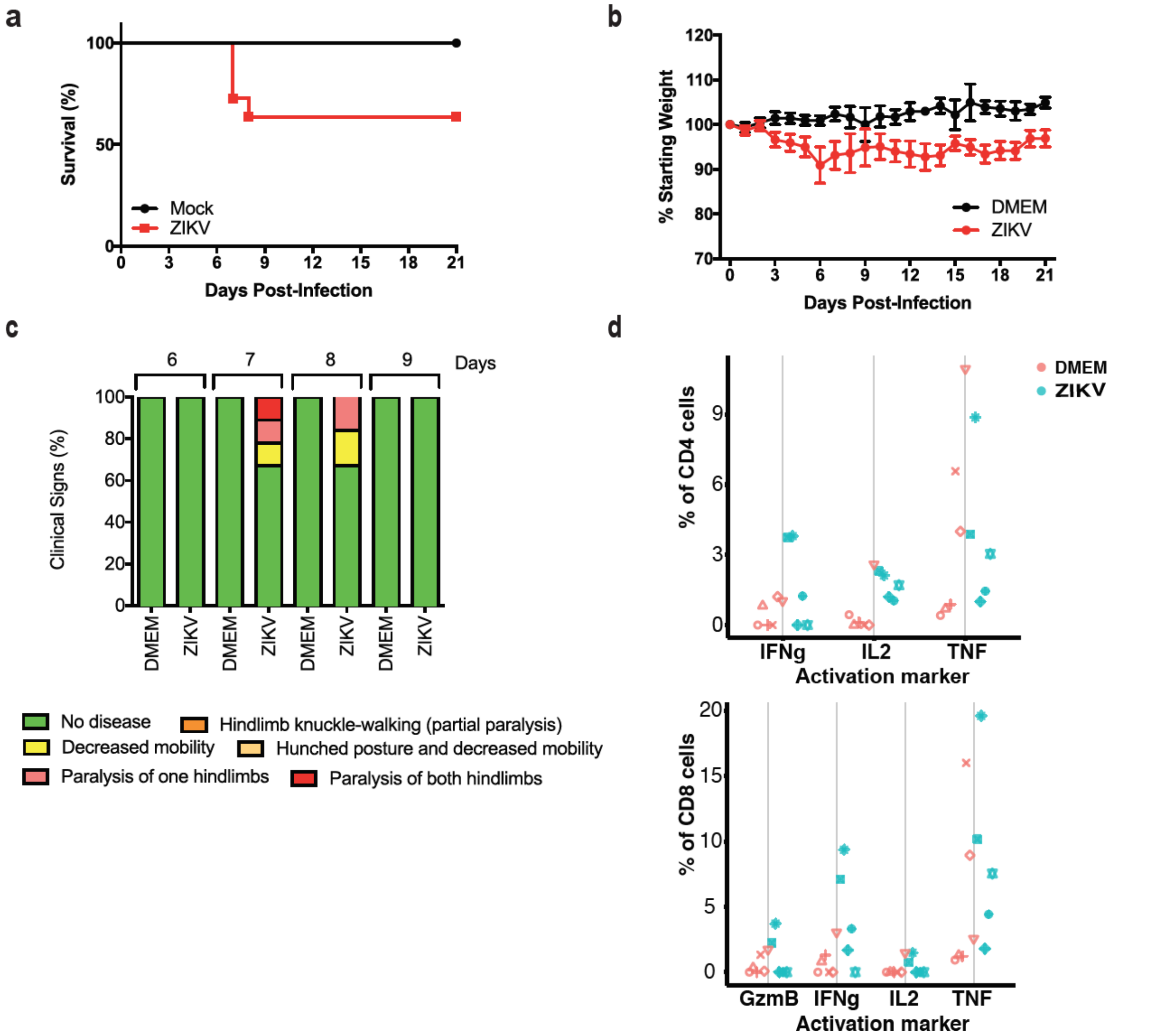

Supplementary Figure 1. ZIKV infection of type-I interferon receptor deficient mice. Ten to eleven week-old male Ifnar1$^{-/-}$ mice were inoculated with 5x10^{5} pfu of ZIKV strain PRVABC59 by a subcutaneous route (s.c.) into each hind leg (1x10^{6} p.f.u. total) and compared to PBS mock-infected mice. Mice were observed daily to assess survival (**a**) weight loss (**b**), and clinical signs (**c**). The percentage of mice from each group presenting the indicated clinical sign is displayed. **d**, Flow cytometric analysis of T-cell responses. Spleenocytes were stimulated with overlapping peptide pools spanning the entire envelope protein of ZIKV. Frequencies of ZIKV peptide specific IFN-γ, IL2, TNF-α, and granzyme B (GzmB) secreting cells were measured by flow cytometry. The data shown are from one experiment (n = 6) that is representative of the same outcome in the two studies performed. Bars indicate mean values and error bars indicate standard deviation. The data shown are for the same mice described in Figure 1.

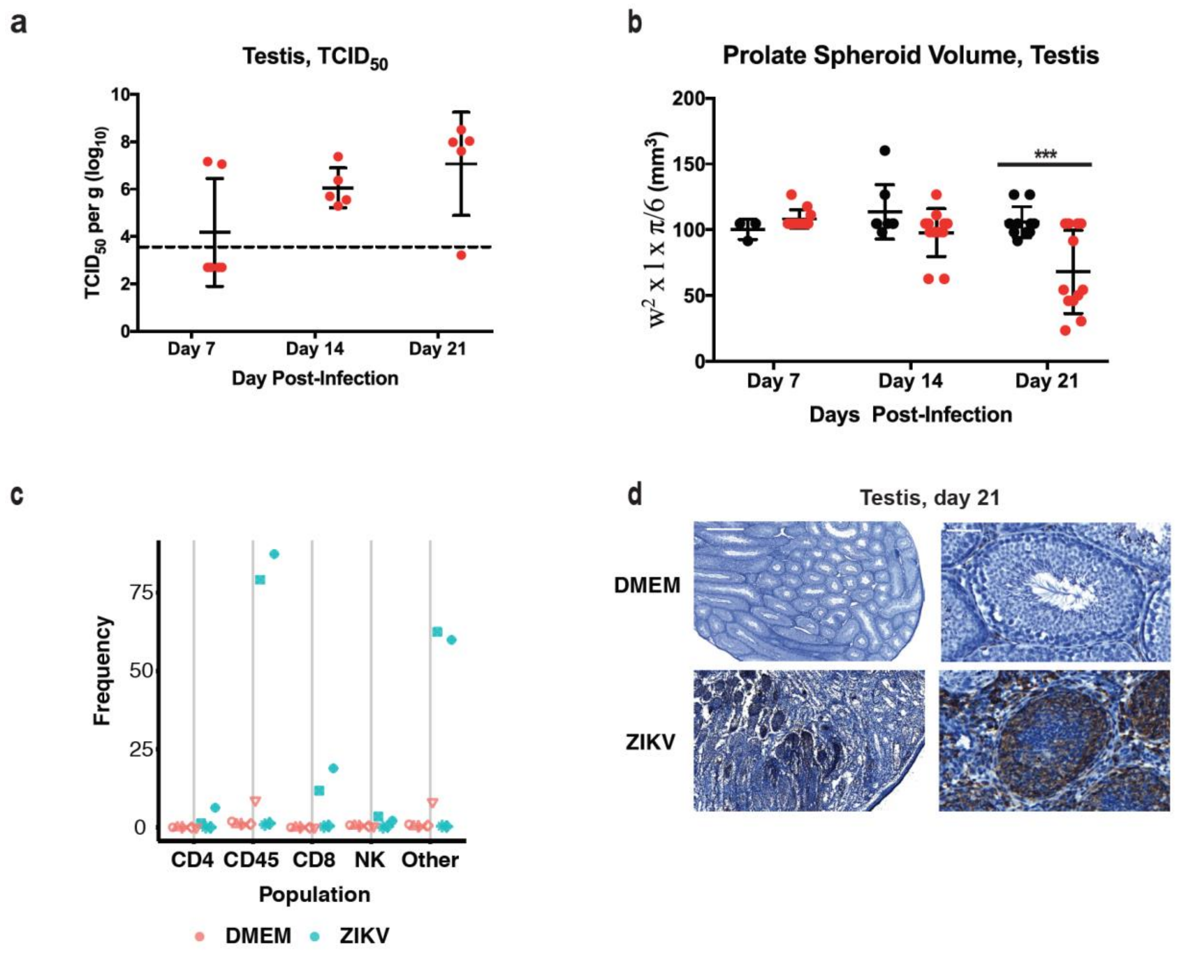

Supplementary Figure 2. ZIKV infection of the testis. Ten to eleven week-old male Ifnar1$^{-/-}$ mice were inoculated with $5x10^5$ pfu of ZIKV strain PRVABC59 by a subcutaneous route (s.c.) into each hind leg ($1x10^6$ p.f.u. total) and compared to PBS mock-infected mice. **a**, Infectious virus titers in homogenized testis tissue measured by tissue culture infective dose ($TCID_{50}$) (n = 5). The horizontal hatched line indicates the limit of detection. **b**, Prolate spheroid volume of the testis based on length and width measurements (mock = black, ZIKV = red). **c**, Immune cell infiltration into the testis at 21 days after infection measured by flow cytometry. **d**, Immunohistochemical analysis of macrophage infiltration into the testes at 21 days after infection. Magnification is 5 x, scale bar 500 µm (left panel) and 40 x, scale bar 50 µm (right panel). Statistical differences are given (two-way ANOVA followed by the Bonferroni post test). *** P<0.001. The data shown are from one experiment (n = 6) that is representative of the same outcome in the two studies performed. Bars indicate mean values and error bars indicate standard deviation. The data shown are for the same mice described in Figure 1.

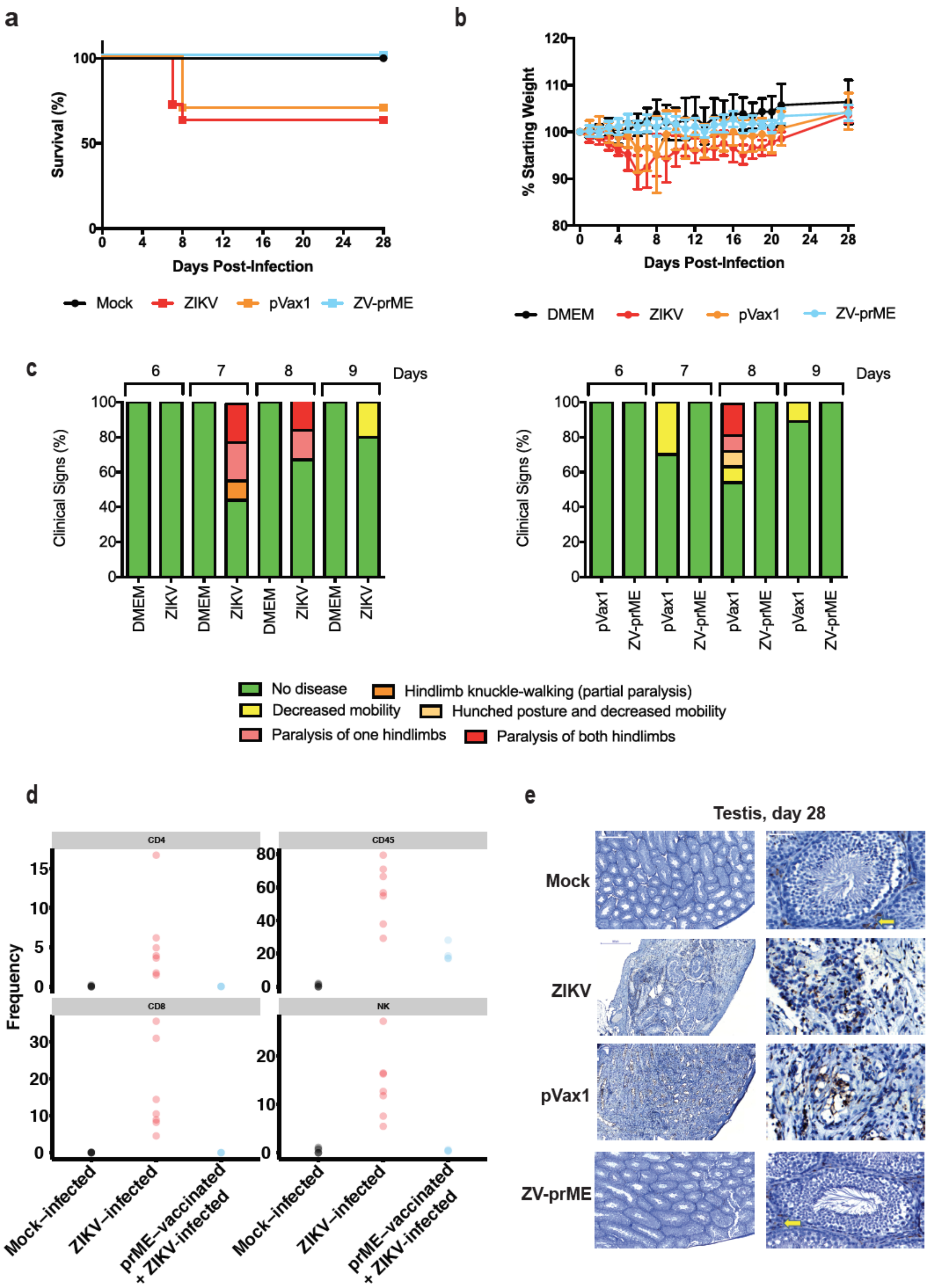
a
Survival (%)
Days Post-Infection
Mock
ZIKV
pVax1
ZV-prME
b
% Starting Weight
Days Post-Infection
DMEM
ZIKV
pVax1
ZV-prME
c
Days
Clinical Signs (%)
DMEM
ZIKV
pVax1
ZV-prME
No disease
Hindlimb knuckle-walking (partial paralysis)
Decreased mobility
Hunched posture and decreased mobility
Paralysis of one hindlimbs
Paralysis of both hindlimbs
d
CD4
CD45
CD8
NK
Frequency
Mock–infected
ZIKV–infected
prME–vaccinated + ZIKV-infected
e
Testis, day 28
Mock
ZIKV
pVax1
ZV-prME

Supplementary Figure 3. ZIKV infection of immunized type-I interferon receptor deficient mice. Five to six week-old male Ifnar1$^{-/-}$ mice received two vaccinations by the i.m. route with electroporation-mediated delivery at 2-week intervals with 25 µg of the control vector (pVax1) or ZIKV-prME DNA vaccine (ZV-prME). Mice were challenged two weeks after the second immunization with a total of $1x10^6$ p.f.u. of ZIKV strain PRVABC59 by a subcutaneous route (s.c.) and compared to age-matched PBS mock-infected (mock) or ZIKV-infected (ZIKV) mice. Mice were observed daily to assess survival (**a**) weight loss (**b**), and clinical signs (**c**). The percentage of mice from each group presenting the indicated clinical sign is displayed. **d**, Immune cell infiltration into the testis at 28 days after infection measured by flow cytometry. **e**, Immunohistochemical analysis of macrophage infiltration into the testes at 28 days after infection. Yellow arrows indicate resident macrophages present in the interstitial space between the seminiferous tubules in healthy mice. Magnification is 5 x, scale bar 500 µm (left panel) and 40 x, scale bar 50 µm (right panel). The data shown are from one experiment (n = 6) that is representative of the same outcome in the two studies performed. Bars indicate mean values and error bars indicate standard deviation. The data shown are for the same mice described in Figure 3.

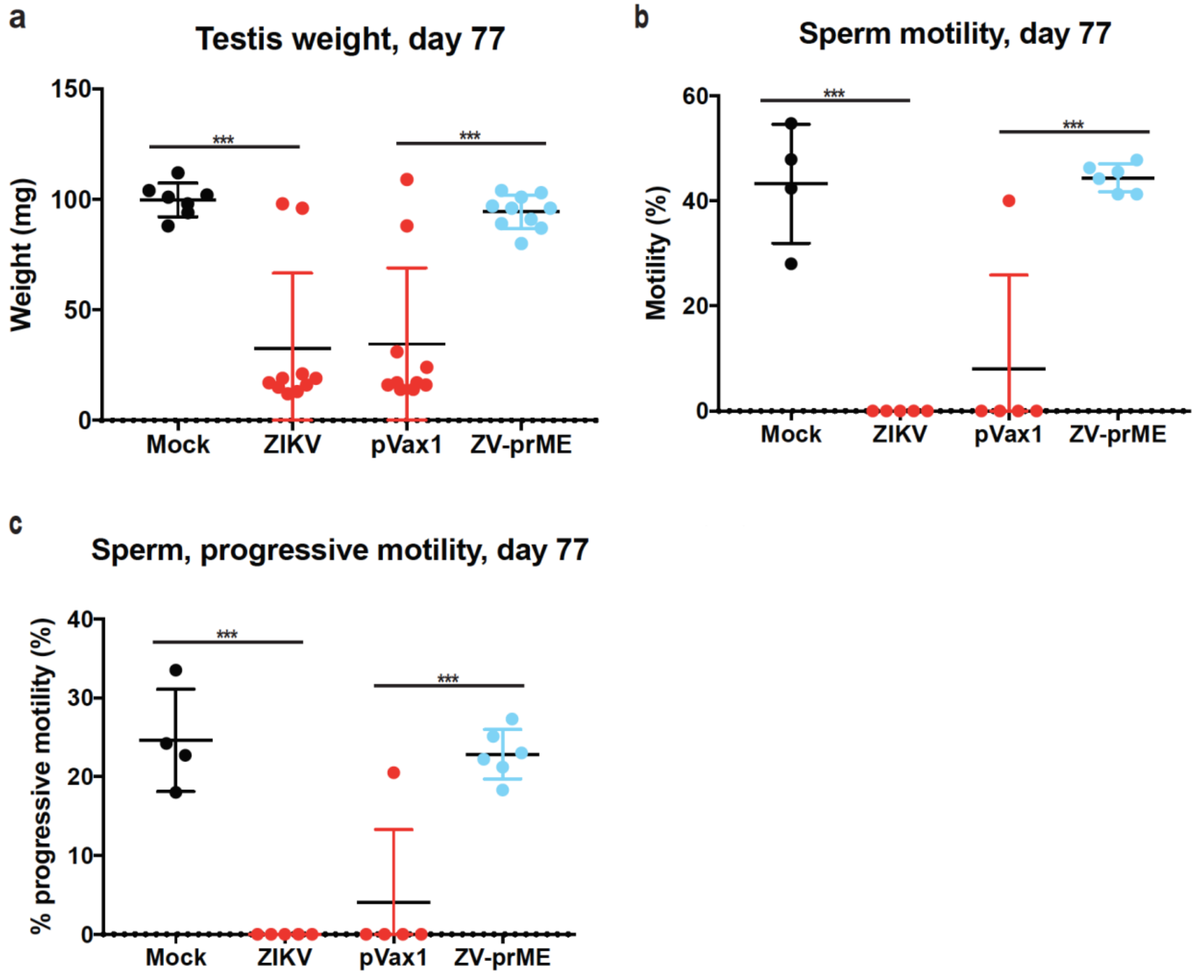

Supplementary Figure 4. ZIKV infection of immunized type-I interferon receptor deficient mice. Five to six week-old male Ifnar1$^{-/-}$ mice received two vaccinations by the i.m. route with electroporation-mediated delivery at 2-week intervals with 25 µg of the control vector (pVax1) or ZIKV-prME DNA vaccine (ZV-prME). Mice were challenged two weeks after the second immunization with a total of 1x10^6 p.f.u. of ZIKV strain PRVABC59 by a subcutaneous route (s.c.) and compared to age-matched PBS mock-infected (mock) or ZIKV-infected (ZIKV) mice **a**, Weight of the testis (2 per mouse). **b, c**, Fertility parameters of caudal sperm were assessed (n = 4 or greater), including percentage motility (**b**) and progressive motility (**c**). The data shown are from one experiment (n = 6) that is representative of the same outcome in the two studies performed. Bars indicate mean values and error bars indicate standard deviation. Statistical differences are given (**a**, two-way ANOVA followed by the Bonferroni posttest **b**, **c**, one-way ANOVA followed by Dunnett's test. ***, $p < 0.001$). The data shown are for the same mice described in Figure 3.
